# Lung Ultrasound Score in Neonatal RDS: Agreement between Sonography Expert and Neonatologists with Varying Levels of Experience

**DOI:** 10.3390/healthcare12141450

**Published:** 2024-07-20

**Authors:** Joanna Puskarz-Gąsowska, Piotr Kruczek, Roman Hożejowski, Małgorzata Stefańska, Witold Błaż, Iwona Sadowska-Krawczenko, Urszula Majewska, Renata Bokiniec

**Affiliations:** 1 Department of Neonatology and Neonatal Intensive Care, Medical University of Warsaw, 00-315 Warsaw, Poland; asiapuskarz@wp.pl (J.P.-G.); ulmaj@wp.pl (U.M.); renata.bokiniec@wum.edu.pl (R.B.); 2 Department of Neonatology, Ujastek Medical Center, 31-752 Cracow, Poland; 3 Department of Neonatology, Czerwiakowski Hospital at Siemiradzki st., 31-137 Cracow, Poland; 4 Medical Department, Chiesi Poland, 02-305 Warsaw, Poland; r.hozejowski@chiesi.com; 5 Neonatal and Intensive Care Department, F. Chopin District Specialist Hospital, 35-055 Rzeszów, Poland; meg0107@interia.pl; 6 Neonatal and Intensive Care Department, Rzeszów Provincial Hospital No. 2, 35-055 Rzeszów, Poland; witekblaz@yahoo.com; 7 Faculty of Medicine, University of Rzeszów, 35-055 Rzeszów, Poland; 8 Department of Neonatology, Jan Biziel University Hospital No. 2, 85-168 Bydgoszcz, Poland; sadowskakrawczenko@gmail.com

**Keywords:** neonate, premature, lung ultrasound score, interrater agreement, respiratory distress syndrome

## Abstract

This study aimed to assess interrater agreement in lung ultrasound scores (LUS) among neonatologists with varying experience levels and an expert sonographer. A post hoc analysis was conducted on data from a prospective multicenter study involving 155 infants born <34 weeks’ gestation, all with respiratory distress syndrome. A total of 629 lung scans were performed and video-recorded by 21 point-of-care sonographers, including both experienced (n = 7) and inexperienced (n = 14) evaluators. Subsequently, a blinded expert sonographer re-evaluated the assigned LUS values. The Cohen’s kappa statistic for individual pulmonary field assessments ranged from 0.89 to 0.93, indicating nearly perfect agreement. The interclass correlation coefficient (ICC) confirmed excellent reliability on total LUS values, demonstrating similar performance of experienced (ICC = 0.92, 95% CI 0.90–0.94) and inexperienced sonographers (ICC = 0.93, 95% CI 0.92–0.94). This study underscores that lung ultrasound is easily learned, and LUS exhibits outstanding reproducibility, irrespective of the sonographer’s level of experience.

## 1. Introduction

Numerous publications have explored the semi-quantitative evaluation of lung ultrasound scans in neonates with respiratory distress syndrome (RDS), utilizing a numerical score referred to as the lung ultrasound score (LUS). The practical applicability of LUS in managing neonatal RDS has been increasingly substantiated in subsequent studies. These studies not only indicate the potential usability of LUS in daily practice but also delineate specific cut-off values for LUS, which demonstrate significant prognostic capabilities for early predictions of crucial outcomes. These outcomes encompass various factors such as the requirement for surfactant [1,2,3,4], ventilation needs [5], readiness for extubation [6,7], or the development of bronchopulmonary dysplasia [8,9,10,11].

However, a critical caveat arises from the fact that, in many of the published studies, a singular experienced sonographer conducted, or at least rated all of the examinations [3,12,13,14]. This impedes the generalizability of the results until it is established that the semi-quantitative lung ultrasound assessment remains reproducible at the point-of-care. In other words, while following a unified methodology, the assigned LUS scores should demonstrate consistency across neonatal centers, but also among sonographers with varying degrees of expertise.

In this analysis, our objective was to evaluate the agreement between LUS determined with a standardized methodology by neonatologists with different degrees of skill and from various neonatal centers, and a sonography expert.

## 2. Materials and Methods

The data utilized for this analysis originated from a prospective, multicenter cohort study on lung ultrasound in preterm infants experiencing respiratory distress syndrome (RDS). The study spanned from May 2021 to April 2022 and involved a cohort of 155 preterm neonates with a gestational age of less than 34 weeks. These infants underwent sequential lung scans, performed from the early post-birth hours until day 7 of life. The primary objective of the study was to evaluate the prognostic significance of the LUS concerning various neonatal outcomes and involved scoring of lung scans from all centers by one study expert. A detailed account of the study’s findings can be found elsewhere [15].

This paper specifically presents a post hoc analysis focusing on the interrater agreement of LUS. The assessment includes LUS values assigned by point-of-care sonographers with varying levels of competence who performed lung scans for this study at the study locations (neonatologist-performed lung ultrasound—NPLUS), as well as a neonatal ultrasound expert who was blinded to the LUSs assigned in study sites. Point-of-care sonographers were neonatology consultants and residents undergoing specialization in neonatology. The study expert was a highly qualified sonographer and neonatal intensivist, holding dual specialties in neonatology and pediatrics, with over 30 years of experience in ultrasonography. Additionally, the expert is a certified trainer with the Polish Ultrasound Society and the author of lung ultrasound courses for neonatologists.

### 2.1. Procedures and Participants

A total of 21 point-of-care sonographers (co-investigators) from 5 study centers conducted a total of 647 lung scans in 155 infants, with 629 scans (97%) successfully captured as video files for subsequent analysis. The initial lung scans were performed on average at 1.6 h after birth (median), with subsequent scans on Day 2, 3, and 7 of life.

For the purpose of analysis, point-of-care sonographers were categorized into two groups based on their experience level: experienced and inexperienced. As per the consensus of the study’s scientific committee, a sonographer was classified as experienced only if they met all of the following criteria: (1) a minimum of 5 years of overall experience as a sonographer, (2) a minimum of 1 year experience in performing lung ultrasound, and (3) completion of a dedicated course on lung ultrasound or holding a license as an ultrasound instructor from the Polish Ultrasound Society.

The sonographers assessed the lung scans according to the grading system described by Szymański et al. [5]. This method assesses the infant’s anterior and posterior lung fields and incorporates an additional grade of “white lung with fluid alveologram” to the original four-grade scale proposed by Brat et al. [16]. Hence, each pulmonary field is scored on a five-grade scale (0 to 4), and the cumulative LUS of all four pulmonary fields ranges from 0 to 16. In the majority of cases, the same physician conducted subsequent ultrasound examinations for the specific patient. All video-recorded lung ultrasound examinations were independently evaluated by a singular expert, who remained blinded to the assigned scores. Interrater agreement was assessed as the consistency between an expert’s evaluation and ratings of point-of-care sonographers, stratified into experienced and inexperienced evaluators.

### 2.2. Statistical Analyses

To evaluate the consistency of assessments assigned to individual pulmonary fields, we utilized Cohen’s kappa statistics. Ranging from −1 to +1, positive values signify agreement exceeding chance, with +1 denoting perfect agreement. We adopted the widely accepted interpretation by Landis and Koch [17], where Cohen’s kappa values between 0.41 and 0.60 indicate moderate agreement, 0.61 to 0.80 signify substantial agreement, and >0.81 suggest almost perfect agreement.

The intra-class correlation coefficient (ICC), particularly suitable for numerical values or variables with multiple rating categories, was employed to assess the consistency in total LUS values assigned by the expert and co-investigators. ICC values range from 0 to 1, where 1 signifies perfect agreement and 0 denotes no agreement. Values between 0.5 and 0.75 indicate moderate reliability, values between 0.75 and 0.9 suggest good reliability, and values greater than 0.90 indicate excellent reliability [18].

In addition to Cohen’s kappa and the ICC, the percentage of absolute agreement was calculated as number of concordant scores divided by a total number of scores.

## 3. Results

The characteristics of the study population were described in detail elsewhere [15]. In short, the study cohort included infants with a median (IQR) gestational age of 32 (30–33) weeks, and a mean (SD) birth weight of 1660 (495) g, mostly born by cesarean section (79%). The baseline characteristics are presented in Table 1.

Of the 21 co-investigators serving as point-of-care sonographers, 7 were classified as experienced and enrolled 73 patients (47%), while the remaining 14 were categorized as inexperienced and enrolled 82 patients (53%). Notably, nearly all experienced sonographers completed a dedicated lung ultrasound course, whereas just over half of the inexperienced sonographers received similar training, with the rest relying solely on “on-the-job” training on lung ultrasound. Both groups of sonographers performed a similar total number of examinations.

A comparative summary of the professional profiles of experienced and inexperienced sonographers is summarized in Table 2.

### Interrater Agreement Measures

The percentage of absolute agreement with the expert on the assessment of individual pulmonary fields was comparable for both experienced and inexperienced evaluators; *p* = 0.268 between the groups (Table 3).

**Figure 1:**
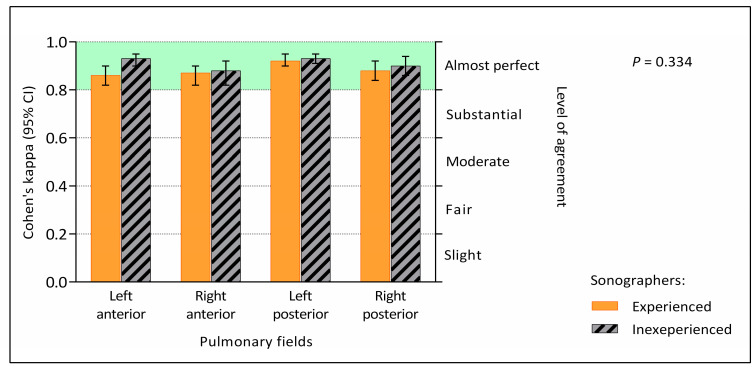
Cohen’s kappa for agreement with expert ratings of individual pulmonary fields. Interpretation of agreement levels according to Landis and Koch [17].

Furthermore, the Kappa statistics confirmed there were no significant differences between the performance of experienced and inexperienced sonographers in evaluation of the lung fields; *p* = 0.334 for between-group comparison (Figure 1).

The cumulative score of all lung fields, referred to as the LUS, showed excellent agreement with expert ratings for both experienced sonographers (ICC = 0.92, 95% CI 0.90–0.94) and inexperienced sonographers (ICC = 0.93, 95% CI 0.92–0.94), with no significant differences observed between the groups.

## 4. Discussion

In the routine use of lung ultrasonography in the neonatal setting, a significant challenge lies in its validation, with a crucial emphasis on ensuring the reliability of assessments (including assigned LUS scores) conducted at the point-of-care. Therefore, in the analysis we specifically investigated how varying levels of experience may impact the quality of lung scan assessments.

The primary message derived from our study is the remarkable ease with which neonatal trainees can acquire sufficient proficiency in assessment of lung scans in infants with RDS. Unlike traditional imaging techniques that often necessitate advanced skills and extensive training, our results indicate that lung ultrasound can be learned effectively within a relatively short period.

In our prior work, we demonstrated a very high level of agreement between two sonography experts in assessing images from premature newborns exhibiting a spectrum of pulmonary conditions—from RDS and congenital pneumonia to bronchopulmonary dysplasia—within one month after birth [5]. In the present study, we confirmed this reproducibility of assessments in a multicenter setting involving a large group (21 doctors) of point-of-care sonographers.

The implications of this key finding are substantial, as it opens doors for a broader implementation of lung ultrasonography across diverse healthcare settings. The repeatability of assessments is a critical requirement for translating the findings of clinical trials into routine practice. In many studies, the analysis of repeatability in lung scan assessments and the assigned scores is often presented in a limited manner or sometimes not addressed altogether [14,19,20,21]. Doubts regarding the repeatability of scan assessments stem from two main concerns. Firstly, there are studies where lung field scans are depicted as single images rather than as videos [3,12]. While scoring freeze-frame images may yield higher interrater consistency, it is important to recognize that video recordings provide more comprehensive information about the severity of changes in specific lung areas. Notably, some publications do not report the type of material analyzed [13,22]. Secondly, the consistency of assessments is often analyzed on a limited number of patients, representing only a small percentage of the total study participants [23]. In our study, however, the assessment of consistency encompassed all the collected scans.

It is important to emphasize that we observed high consistency between expert assessments and those performed by point-of-care sonographers, despite the fact that the expert evaluated recorded video material rather than conducting bedside examinations himself. This method of assessment posed additional challenges, as the recorded sequences were typically 8–10 s in length and represented only a limited portion of the complete ultrasound examination. The attainment of high agreement using this methodology is particularly valuable and underscores the robustness of our findings.

In the realm of ultrasound, particularly in lung examinations, there are often discussions about the subjectivity involved in the assessment process. However, critics overlook the fact that the interpretation of other imaging tests, such as computed tomography scans, is also inherently subjective [24].

Based on the very high degree of agreement between expert LUS assessments and those of doctors with varying levels of experience, our study indicates that the fundamental requirement for the successful application of the LUS scale in standard clinical practice is met. The steep learning curve and very good performance of relative novices in ultrasonography, as observed in our study, have significant implications for medical education and healthcare practice. Incorporating lung ultrasonography into training curricula can enhance the diagnostic capabilities of healthcare professionals without imposing a burdensome learning process. This accessibility may prove particularly valuable in resource-limited settings where advanced imaging facilities may be scarce.

The ease of learning lung ultrasonography has the potential to democratize diagnostic capabilities across various specialties, enabling a broader range of healthcare professionals to integrate this tool into their clinical assessments. This democratization aligns with the current trend towards point-of-care diagnostics, allowing for quicker and more informed decision-making at the bedside. In this context, it is noteworthy to mention the call for adopting a universal scale for lung assessment [25]. A recommended practice would be to present the lung scan results as a percentage of the maximum value of the scale used [7]. This approach enhances standardization and facilitates comparison across studies and clinical settings.

## 5. Conclusions

Our study underscores the user-friendly nature of lung ultrasonography, emphasizing its potential to become an integral part of routine clinical practice. The simplicity of the learning process, combined with the diagnostic accuracy demonstrated by our participants, positions lung ultrasonography as a promising and accessible tool in daily neonatal practice. Future research could explore the broader implications of widespread adoption and the specific clinical scenarios in which lung ultrasonography can have the greatest impact.

## Figures and Tables

**Table 1:** Descriptive statistics of the study cohort.

	n = 155
Gestational age (weeks), median (IQR)	32 (30–33)
Gestational age categories, n (%)	
24–28 weeks	22 (14)
29–32 weeks	78 (50)
33–34 weeks	55 (36)
Birth weight (g), mean ± SD	1660 ± 495
Sex (male), n (%)	93 (60)
Cesarean section, n (%)	123 (79)
5 min Apgar, median (IQR)	8 (4–10)
Lung Ultrasound Score (LUS), median (IQR)	
0–6 h from birth	5 (2–8)
Day 2	1 (0–6)
Day 3	1 (0–5)
Day 7	1 (0–2)

**Table 2:** Comparison of the professional experience among the point-of-care sonographers.

	Sonographers’ Classification	
	Experienced n = 7	Inexperienced n = 14
Total number of scans evaluated	310	319
Professional qualifications, n (%)		
Consultant neonatologist	5 (71)	5 (36)
Resident in neonatal medicine	2 (29)	9 (64)
Overall experience in ultrasonography, n (%)		
<1 year	0 (0)	2 (14)
1–3 years	0 (0)	7 (50)
3–5 years	2 (29)	5 (36)
>5 years	5 (71)	0 (0)
Experience in lung ultrasound, n (%)		
<1 year	0 (0)	6 (43)
1–3 years	6 (86)	6 (43)
3–4 years	0 (0)	2 (14)
≥5 years	1 (14)	0 (0)
Course on lung ultrasound completed, n (%)	6 (86)	8 (57)
Certified instructor of the Polish Ultrasound Society, n (%)	1 (14)	0 (0)

**Table 3:** Measures of agreement with expert ratings, stratified by sonographers’ experience.

Scored Area	No. of Scans	No. of Concordant Scores	Percent Absolute Agreement	Weighted Cohen’s Kappa		Interclass Correlation Coefficient	
				Value	95% CI	Value	95% CI
	Experienced sonographer						
Left anterior	310	258	83.2	0.86	0.82–0.90	–	–
Right anterior	310	265	85.5	0.87	0.82–0.90	–	–
Left posterior	310	265	85.5	0.92	0.90–0.95	–	–
Right posterior	310	253	81.6	0.88	0.84–0.92	–	–
Total LUS score	310	212	68.4	–	–	0.92	0.90–0.94
	Inexperienced sonographer						
Left anterior	319	281	88.1	0.93	0.90–0.95	–	–
Right anterior	319	279	87.5	0.88	0.82–0.92	–	–
Left posterior	319	282	88.4	0.93	0.91–0.95	–	–
Right posterior	319	273	85.6	0.90	0.86–0.94	–	–
Total LUS score	319	232	72.7	–	–	0.93	0.92–0.94

## Data Availability

The datasets analyzed during the current study are available from the corresponding author on reasonable request.
